# Context conditioning in humans using commercially available immersive Virtual Reality

**DOI:** 10.1038/s41598-017-08184-7

**Published:** 2017-08-17

**Authors:** Marijn C. W. Kroes, Joseph E. Dunsmoor, Wayne E. Mackey, Mason McClay, Elizabeth A. Phelps

**Affiliations:** 1 New York University, Department of Psychology, New York, NY 10003 USA; 2 New York University, Center for Neural Science, New York, NY 10003 USA; 3 University of Texas at Austin, Department of Psychiatry, Austin, TX 78712 USA; 4 Centre College, Department of Psychology, Danville, KY 40422 USA; 5 Nathan Kline Institute, Orangeburg, NY 10962 USA

## Abstract

Despite a wealth of knowledge on how humans and nonhuman animals learn to associate meaningful events with cues in the environment, far less is known about how humans learn to associate these events with the environment itself. Progress on understanding spatiotemporal contextual processes in humans has been slow in large measure by the methodological constraint of generating and manipulating immersive spatial environments in well-controlled laboratory settings. Fortunately, immersive Virtual Reality (iVR) technology has improved appreciably and affords a relatively straightforward methodology to investigate the role of context on learning, memory, and emotion while maintaining experimental control. Here, we review context conditioning literature in humans and describe challenges to study contextual learning in humans. We then provide details for a novel context threat (fear) conditioning paradigm in humans using a commercially available VR headset and a cross-platform game engine. This paradigm resulted in the acquisition of subjective threat, threat-conditioned defensive responses, and explicit threat memory. We make the paradigm publicly available and describe obstacles and solutions to optimize future studies of context conditioning using iVR. As computer technology advances to replicate the sensation of realistic environments, there are increasing opportunities to bridge the translational gap between rodent and human research on how context modulates cognition, which may ultimately lead to more optimal treatment strategies for anxiety- and stress-related disorders.

## Introduction

Contextual information plays an important role in the development, maintenance, and treatment of anxiety and stress related disorders^1–3^. Much of what we know about the role of context in emotional learning and memory is informed by Pavlovian conditioning research, predominately in rodents. In context conditioning experiments, for instance, the subject learns to associate the presentation of a salient outcome, such as an electrical shock or food, with the environment it is in — typically the conditioning chamber — rather than with a discrete cue like a tone or a light. Behavioural and neurophysiological research on context conditioning in laboratory animals has increased our understanding of how the brain codes contextual representations and stores memories of threatening and rewarding environments. However, unlike Pavlovian conditioning that involves discrete cues, the translation of context conditioning research from rodents to humans has fallen short. This is in large measure due to a practical limitation; specifically, what is the human analogue to the rodent conditioning chamber? Posed another way: how does the experimenter effectively develop and incorporate complex environments composed of multiple spatiotemporal and sensory features while maintaining strong experimental control? As Pavlovian learning is thought to contribute to anxiety and stress disorders^4–6^, overcoming this limitation could contribute to more ecologically valid research on how contextual processes contribute to psychopathology. In the present article, we provide an overview of context conditioning research in the domain of threat (fear) learning, discuss practical and theoretical limitations to some of the existing context conditioning protocols in humans, and describe methods and results from a new threat context conditioning protocol using immersive virtual reality (iVR) that address those limitations.

Pavlovian conditioning provides a valuable laboratory model to investigate the acquisition, expression, generalization, and inhibition of threat-related behaviour across species^4–6^. Two Pavlovian conditioning paradigms have served as the workhorses to understand the mechanisms underlying learned threat and the role of contextual processes: cue conditioning and context conditioning. In standard aversive Pavlovian *cue conditioning* a subject learns that a neutral stimulus in the environment (conditioned stimulus, CS, e.g. a tone or picture) predicts the occurrence of an aversive outcome (unconditioned stimulus, US, e.g. an electrical shock). Once the subject learns the CS-US association, presentations of the CS alone begin to elicit threat-related defensive responses, such as freezing and increases in sympathetic arousal. Cue conditioning methodology is easily translatable from laboratory animals to humans, where simple sensory cues like tones and lights or more complex stimuli like faces and object categories can serve as CSs. Pavlovian threat conditioning continues to serve as a valuable and tractable model for psychopathologies characterized by acute or phasic threat responses triggered by discrete cues; exemplified by Obsessive Compulsive Disorder, Phobias, and symptom clusters of PTSD that involve defensive reactions to trauma-reminders [see ref. 4 for review].

In Pavlovian *context conditioning*, the US is delivered while the subject is in a particular environment (i.e., the conditioning chamber) but is not signalled by a discrete CS. Notably, the term ‘context’ in associative learning research can be broadly defined, ranging from the sensory details of the environment (sights, smells, sounds, etc.), to internal states, to perceptions of time^7^. Context conditioning provides a more suitable model for psychopathologies characterized by sustained or ‘free-floating’ anxiety when there is no clear threat-eliciting stimulus in the environment^8^; exemplified by Generalized Anxiety Disorder or symptom clusters of PTSD that involve hyper-arousal in the absence of threat-eliciting cues.

Context also interacts with cue conditioning to modulate expression of the CS-US associative memory. For example, a CS that predicts the US in one context might then be presented without the US in a separate context, i.e., extinction. Testing the CS in the first (i.e., acquisition), second (i.e., extinction context), or a novel context can reveal which CS association the subject retrieves. Neurobehavioral research has shown how context gates expression of CS-US associative memories^7, 9^; for example, cue-elicited responses may be reduced at a later test in the extinction context (extinction recall) or may re-emerge in the extinction context (spontaneous recovery) or in the acquisition or novel context (renewal). Notably, the present article focuses largely on context conditioning paradigms where only the spatiotemporal environment signals the US in the absence of a discrete threat-eliciting cue. In human literature the term context conditioning has often been misapplied to describe paradigms where context modulates cue conditioning. We will explain the importance of distinguishing context conditioning and context modulated cue conditioning below.

Neurophysiological research in laboratory animals on context threat conditioning has focused predominately on the interplay between the amygdala, hippocampus, bed nucleus of the stria terminalis (BNST) and medial prefrontal cortex^7, 9–12^. While a comprehensive review of the neurobiology of context conditioning is beyond the scope of the present article, the amygdala is considered the critical site for the formation of CS-US associations and the expression of phasic learned defensive responses during aversive cue conditioning^13, 14^. The hippocampus has a broad role in contextual processes - including spatial representations and navigation^15^ – contributing to the acquisition and storage of contextual threat information^13, 14, 16, 17^. The hippocampus also interacts with the bed nucleus of the stria terminalis (BNST) to sustain defensive responses to diffuse threats^18–19^, and interacts with the medial prefrontal cortex during the inhibition of threat responses in safe environments^21–23^.

Despite the increasing number of studies on contextual processes in laboratory animals, there are to date few systematic investigations of context conditioning in humans (Table 1). This is largely due to the challenge of creating a spatiotemporal context to pair with a US. In laboratory animals, context is manipulated by changing the animal’s physical location (e.g., a different conditioning box) or space (e.g., change in the colours of the walls, texture or the floor and/or smell). The most obvious analogue to the conditioning chamber for human subjects is the laboratory testing room. Although the room is the predominant context, it is often not the target of context conditioning. In other words, unlike in laboratory animal research, in human conditioning studies the experimenter generally does not intend for the human subject to form an association between the physical testing room environment (composed of particular lighting, furniture, computers, research equipment, etc.) and the US.

**Table 1:** Literature overview context conditioning in humans.

Publication	Paradigm	Context manipulation	Measures: During task			US exp.	fMRI	Before and after task		Anxiety	STAI-S	After task	
			FPS	SCR	SCL			Arousal	Affect/Valence			PANAS	Contingency
Kroes *et al*., current submission	CTX	iVR, 1a	✓	✓				✓	✓				✓
Troger *et al*.^74^.*	CTX	iVR, 2b	✓	✓				✓	✓	✓	✓	✓	✓
Glotzbach^59^	CTX	iVR, 2b	✓		✓			✓	✓	✓			✓
Glotzbach-Schoon *et al*.^56^	CTX	iVR, 2b	✓		✓					✓			✓
Glotzbach-Schoon *et al*.^92^	CTX	iVR, 2b	✓		✓					✓	✓	✓	✓
Glotzbach-Schoon *et al*.^58^	CTX	iVR, 2b	✓		✓	✓				✓	✓	✓	✓
Andreatta *et al*.^40^	CTX	iVR, 2b&c					✓			✓	✓	✓	✓
Andreatta *et al*.^48^	CTX	iVR, 2b	✓		✓			✓	✓	✓		✓	✓
LaBar *et al*.^24^	Cue in CTX	Test room		✓									
Neuman *et al*.^26^	Cue in CTX	Test room				✓							
Schiller *et al*.^44^	Cue in CTX	Test room		✓									
Huff *et al*.^27^	Cue in CTX	Test room		✓									
Huff *et al*.^35^	Cue in CTX	Full iVR, 3		✓		✓							
Muhlberger *et al*.^75^	Cue in CTX	iVR, 2b	✓							✓			
Dunsmoor *et al*.^37^	Cue in CTX	VR, 3b	✓										
Ahs, *et al*.^39^	Cue in CTX	VR, 3c		✓		✓				✓			
Baas *et al*.^30^	Cue in CTX	2D Movie	✓							✓			✓
Baas *et al*.^76^	Cue in CTX	2D Movie				✓				✓			✓
Grillon *et al*.^77^	Cue in CTX	2D Movie	✓							✓			
Alvarez *et al*.^41^	Cue in CTX	2D Movie	✓	✓						✓			
Alvarez *et al*., 2008^94^	Cue in CTX	2D Movie	✓										
Grillon *et al*., 2008^95^	Cue in CTX	2D Movie	✓										
Armony *et al*.^28^	Cue in CTX	2D Static		✓			✓						
Milad *et al*.^29^	Cue in CTX	2D Static		✓									
Kalisch *et al*.^78^	Cue in CTX	2D Static		✓			✓						
Milad *et al*.^43^	Cue in CTX	2D Static		✓			✓						
Marschner *et al*.^79^	Cue in CTX	2D Static		✓		✓	✓						
Neumann *et al*.^80^	Cue in CTX	2D Static		✓		✓							
Pace-Schott *et al*.^81^	Cue in CTX	2D Static		✓									
Rougemont-Bucking *et al*.^82^	Cue in CTX	2D Static		✓			✓						
Van Ast *et al*.^83^	Cue in CTX	2D Static	✓	✓		✓			✓			✓	
Balooch *et al*.^84^	Cue in CTX	2D Static	✓			✓							
Haaker *et al*.^93^	Cue in CTX	2D Static		✓		✓	✓						
Haaker *et al*.^86^***	Cue in CTX	2D Static	✓	✓									✓
Londsdorf *et al*., 2014^94^	Cue in CTX	2D Static	✓			✓							
Glenn *et al*., 2014^98^	Cue in CTX	2D Static	✓			✓		✓					
Londsdorf *et al*., 2015^85^	Cue in CTX	2D Static	✓		✓	✓							
Kastner *et al*.^95^**	Cue in CTX	2D Static						✓	✓	✓			
Kroes *et al*.^45^	Cue in CTX	2D Static		✓			✓						✓
Haaker *et al*.^86^	Cue in CTX	2D Static		✓		✓							
Sjouwerman *et al*.^87^	Cue in CTX	2D Static	✓	✓				✓					✓
Hermann *et al*.^88^	Cue in CTX	2D Static		✓			✓						
Kuhn *et al*.^89^	Cue in CTX-ITI	2D Static	✓	✓	✓		✓		✓	✓			
Ameli *et al*.^90^	Cue in CTX-ITI	N/A	✓					✓	✓		✓		
Grillon *et al*.^91^	Cue in CTX-ITI	N/A	✓		✓			✓	✓				
Grillon *et al*.^73^	Cue in CTX-ITI	N/A	✓							✓			

Paradigm: CTX = context conditioning, conditioning paradigm where no other cues signal the onset of the unconditioned stimulus. Cue in CTX = cue in context, paradigm where specific cues signal the onset of the unconditioned stimulus dependent on the context in which they occur. Cue in CTX-ITI = Cue in context-inter trial interval, paradigm where specific cues signal the onset of the unconditioned stimulus and defensive responses during inter-trial intervals are taken as a index for context conditioned responses. Context manipulation: iVR = Immersive virtual reality, context manipulation is achieved using head-mounted virtual reality display where head movements are translated into changes in field of view. Test room = context manipulation is achieved by physically moving participants and testing them in different lab spaces. Full iVR = Full immersive Virtual Reality, context manipulation is achieved within room-sized cube (CAVE-like) where the environment is projected onto the walls, floor and ceiling and head movements are translated into changes in field of view. 2D movie = context manipulation is design in 3D but presented as a movie on a 2D computer screen or projected on a screen. 2D static = context manipulation is achieved by presenting a background image. N/A = not applicable, these studies do not manipulate context but take responses during inter-trial intervals as an index of context conditioned responses. *This study also measured heart rate during the task. **This study also measured EEG during the task. ***This study also measured fear ratings during the task. +This study assessed responses during inter-trial-intervals as a proxy for context conditioned responses. FPS = fear potentiated startle; SCR = skin conductance responses; SCL = skin conductance level; US exp. = unconditioned stimulus expectancy ratings; fMRI = functional magnetic resonance imaging; STAI-S = State-Trait Anxiety Inventory-S; PANAS – Positive Affect Negative Affect Scale. References: 24–30, 35, 38–40, 46, 48, 58, 59, 72, 74–95. We apologize for any possible mistakes in our assessment of publications or omission of literature.

One reason the testing room itself is not the target of context conditioning is that human conditioning research includes a within-subject control condition, the CS- (i.e. a stimulus not associated with an aversive outcome), to evaluate non-associative arousal induced by the US (i.e., sensitization). Thus, expression of threat-related behaviour in the shocked testing room (CTX+) would need to be compared to behaviour in another testing room (CTX−). Efforts have been made to create unique physical environments to investigate contextual modulation of cue conditioning^24–27^, but to our knowledge no studies have investigated context conditioning in physical locations, i.e. where the conditioned response is elicited by the context itself.

Investigation in humans on contextual influences on conditioning have instead manipulated context by presenting the CS on top of different static background images or embedded in movies. Such contextual CSs have included simple background colours [e.g. ref. 28], static images of a scene (e.g., living room or office^29^), and first person movies of different environments (e.g., an office and magazine store^30^). Contextual CSs offer important advantages. For instance, the experimenter can counterbalance presentation of the threatening contextual CS and the within-subject control condition, and can control stimulus timing to service analysis of threat-related responses including fear-potentiated startle and tonic arousal indexed by changes in skin conductance levels. However, because contextual CSs are similar in many respects to CSs used in cue conditioning, it raises the question of whether these paradigms involve contextual learning processes, per se.

This distinction between context and contextual CSs is important because contexts can serve multiple functions in Pavlovian conditioning^31^. A context can be the target of conditioning (context conditioning) or modulate cue conditioning. When the context modulates the effectiveness of a cue, but does not elicit a response alone, it is referred to as an ‘occasion setter’^32^. As a practical matter, the testing room is very likely the prevailing physical context. The question therefore arises what it means for a contextual learning system to experience contextual CSs (e.g. background images on a computer screen) within an overriding context (e.g. the test room, or even the entire university building). Are these types of stimuli also processed as ‘contexts’ that recruit hippocampal-dependent learning systems [see ref. 33], or is an amygdala-dependent learning system sufficient for associative threat learning^14^?

Virtual reality technology can be used to optimize contextual stimuli and increase the chance that context conditioning engages contextual learning systems in the brain. Foremost, VR can induce a strong sense of ‘presence’ where people think, behave, feel, and have the sense of being in the virtual space rather than the real world^34^. A critical feature of VR is the headset (or head mounted display), which helps reduce sensory input from the outside environment and in addition allows head movements to be translated into visual rotations creating motion parallax for the participant, thereby increasing the feeling of being engrossed in a virtual environment and removed from the present physical location — referred to as ‘immersion.’ To date, only a handful of context conditioning studies have incorporated VR headsets (Table 1). VR has not yet been in wide use simply because early systems were costly and in some cases required large amounts of space e.g. [ref. 35]. With the commercial release of VR headsets, the ability to augment context conditioning research is now within reach of nearly any research laboratory already investigating human conditioning with psychophysiological equipment. Accessible and validated protocols for commercially available VR systems will prove increasingly useful to the investigation of context conditioning in humans.

The goal of the present study was to develop and test a context conditioning protocol using the commercially available Oculus Rift (Oculus VR) head mounted display and the cross-platform game engine Unity (Unity Technologies, www.unity3D.com). We report the methods and results from this study, and describe obstacles and potential solutions to study learning, memory, and emotion in iVR. Additional details on the protocol and ancillary results are presented in the Supplemental Materials. The protocol and all code are made freely available to the scientific community (https://github.com/wemackey/Kroes_etal_VR). Our results indicate that the iVR context conditioning protocol was well tolerated and resulted in the acquisition of subjective threat, threat-conditioned defensive responses, and explicit threat memory for the aversive context. The development of a freely available context conditioning paradigm using a relatively affordable VR headset and free game engine with an extensive online community is a practical advancement that places the investigation of contextual processes in humans within reach of many laboratories.

## Materials and Methods

### Pilot studies

The context conditioning protocol was extensively piloted to optimize subject comfort and to test how to best maintain experimental control within immersive VR environments. One critical question during pilot testing was whether to allow volunteers to control first-person movement in a virtual environment themselves using a video game controller, or to remove control of movement from the subject and passively guide them through the environment. Pilot testing revealed that allowing subjects to control their own movement throughout the entire context conditioning task created motion-induced nausea in a number of volunteers. Participants with little or no video game experience had difficulty overall orienting to the controller settings during the task, since the VR headset occluded a view of their hands. In addition, some volunteers were also preoccupied with the navigational aspect of the VR environment at the cost of attending to the shock contingencies. This was revealed at debriefing, when a few of the subjects in the pilot experiment could not describe which room the shock occurred in, described being shocked in a room where they never received a shock, or misattributed the shock to an idiosyncratic behaviour, e.g., whatever they happened to be doing at the time, such as looking closely at an item in a room. For the main experiment reported here we therefore decided on allowing subjects to freely navigate and explore the environment for 2 minutes before the context conditioning task, but the subjects did not have control of navigation during the context conditioning task. This ensured that participants could initially form a representation of the environments while having free movement and prevented navigation to be a source of distraction during the actual conditioning task. The limitation of movement control was effective as all but one participant in the main experiment could explicitly indicate the relationship between the rooms and shock correctly at the end of the study (see below). Further details on piloting are provided in the Supplemental Materials.

### Participants

Volunteers for the final version of the task were twenty-two adult volunteers (10 female; Mean Age ± SD: 22.32 ± 4.7; range 18–35). Eligibility requirements were no psychiatric or neurological history, no medication (with the exception of paracetamol or ibuprofen) in the 72 hours prior to the experiment, no consumption of alcohol 24 hours before the experiment, normal or corrected-to-normal vision, normal hearing, and not easily prone to motion sickness. The study was approved by the University Committee on Activities Involving Human Subjects at New York University. All participants provided written informed consent. All methods were carried out in accordance with the Declaration of Helsinki.

### Unconditioned Stimulus (US)

The electrical shock was a 200 millisecond pulse delivered to the right wrist using disposable pre-gelled electrodes connected to a Grass Medical Instruments stimulator (Warwick, RI). Shocks were calibrated using an ascending staircase procedure starting with a low voltage setting near a perceptible threshold and increasing to a level deemed “maximally uncomfortable but not painful” by the participant, in keeping with prior threat conditioning protocols from our laboratory (e.g. refs 36, 37).

### VR and auditory equipment

**Figure 1:**
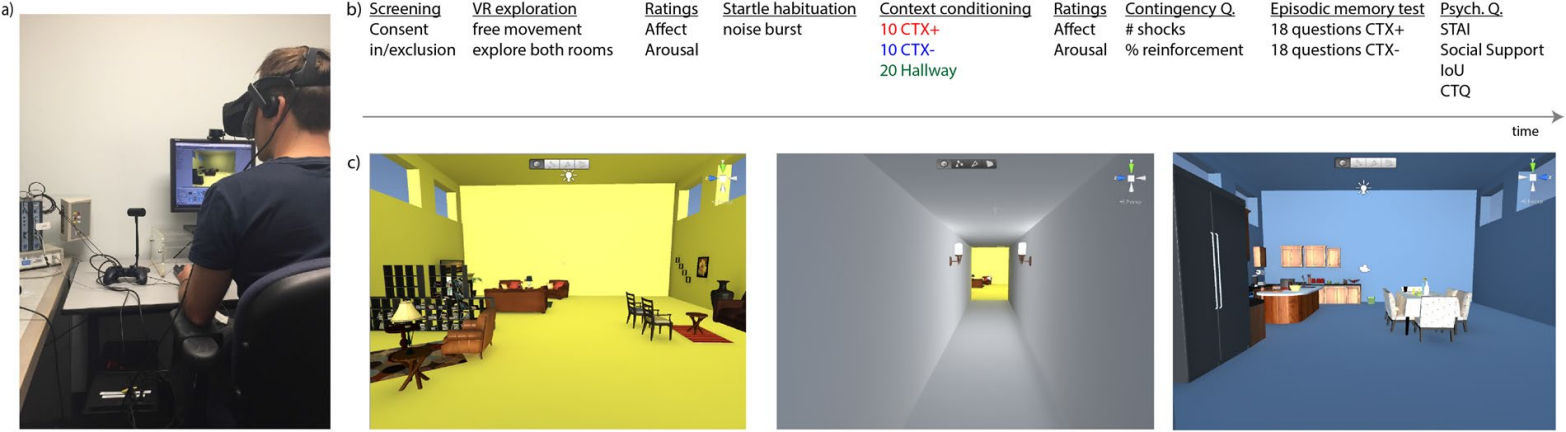
iVR study design. (**a**) iVR experimental set-up showing a participant (not an actual participant) wearing the Oculus Rift head mounted display while the context conditioning paradigm is presented with Unity on a standard desktop and electromyography and skin conductance responses are simultaneously recorded. (**b**) Time-line of experimental design. (**c**) 2D depiction of the two rooms and the hallway in the iVR environment (note that control buttons at the top and top right of the screen were not visible to the participants).

Participants wore the consumer version of the Oculus Rift headset throughout the context conditioning task (Fig. 1). The Oculus Rift displays stereoscopic 3D images at 106.19 degrees horizontal and 95.06 degrees vertical (i.e. 100 degrees diagonal) running at 90 Hz. The headset has a positional tracking system that allows subjects to have full 360**°** movement. The Oculus Rift allows for head-movements to be translated into rotations in the field of view creating motion parallax for the participant. We removed the headphone component of the Oculus rift headset and subjects wore Sennheiser HD280 headphones. These headphones fully covered the ear and therefore provided better control of sound presentation for the short static auditory bursts of white noise used to evoke eye-blink startle responses.

### Eye-blink startle

The eye-blink startle response is a defensive reflex to the presentation of salient stimuli often measured by electromyography (EMG) of the muscles of the eye. Startle has been used in both cue- and context conditioning studies in humans and provides a reliable and valid measure of conditioned learning. Startle responses can be elicited by the sudden presentation of brief salient stimuli, like loud noises, but the response tends to be enhanced under threat, referred to as ‘fear-potentiated startle’. We measured EMG of the right orbicularis oculi muscle at 1000 Hz using two cup electrodes filled with electrolyte gel. A ground electrode was attached to the right hand. Startle probes were binaural bursts of 100 dB white noise presented for 50 milliseconds. Responses to the startle probe were quantified as the maximum EMG response 20–120 ms after the onset of the startle probe, subtracting a baseline measure of the mean EMG magnitude in a 500 ms period prior to the onset of the probe. Startle responses were transformed to *T*-Scores (*z*-score*10 + 50) as in prior studies of cue^38, 3s^ and context conditioning^40, 41^. Before the context conditioning experiment began, subjects listened to 9 startle probes while viewing a blank grey screen (without the VR headset) to allow startle responses to habituate.

### Skin conductance

Skin conductance was measured with pre-gelled snap electrodes (BIOPAC EL509) placed on the hypothenar eminence of the palmar surface of the non-dominant hand. Data were collected using BIOPAC MP-100 System (Goleta, CA), and analysed using an in-house analysis program written in Matlab (the MathWorks) using FieldTrip^42^. Data were low-pass filtered at 5 Hz. Responses to startle probes and upon transitions into the different contexts were determined as the peak-to-peak amplitude difference in skin conductance of the largest deflection in the latency window from 0–4.9 s after event onset (see results) to ensure that responses could not be contaminated by other events (e.g. the shock or following startle probes). The raw skin conductance responses were square root transformed, in keeping with previous studies [e.g. refs 43–47].

### Virtual Contexts

The Virtual Reality environment was designed in Unity 5 (Unity Technologies, www.unity3d.com). A C^#^ script was used in Unity to send TTL pulses in order to trigger electrical shocks and record event timing in peripheral devices (i.e., BIOPAC® Systems Inc.). The unity scripts and assets are available on : https://github.com/wemackey/Kroes_etal_VR). The environment consisted of a virtual living room and kitchen/dining room connected via a hallway (Fig. 1). One room had yellow walls, ceiling and floor and was decorated as a living room. The other room had blue walls, ceiling and floor and was decorated as a kitchen with dining area. These rooms were designed to be highly discriminable so as to prevent generalization of contextual threat learning^48^. Before context conditioning, we allowed subjects to freely explore the rooms with a game controller. This allowed subjects to encode a representation of the environment prior to conditioning, which animal research shows is critical to acquisition of threat to the context [see ref. 49].

During the context conditioning task, participants were passively guided through the two rooms on a continuous predefined path. Subjects had free range of head movement and rotation of the field of view, but were asked to remain mostly still to reduce motion artefacts to the physiological equipment. The path started in the hallway then led through a room, back to the hallway, into a room, back to the hallway, etc. Subjects travelled through each room 10 times for 30 seconds each. Each trip through the hallway (20 total) lasted 15 seconds. We created unique paths so that subjects experienced subtly different trips through the rooms throughout the experiment. On 4 trips through the hallway, the path turned back 180 degrees and subjects returned back to the room they had just exited. The purpose of these ‘return trips’ was to prevent participants from predicting the next room with complete certainty. Such pseudo-randomization of CS trials is routine in differential conditioning research. Noise probes were presented in both rooms and the hallway. Shocks were only administered in one room (CTX+) but not in the other room (CTX−) or the hallway. We pseudo-randomized the order of the startle probes and shocks for different paths to mitigate temporal prediction of the US based on the startle probes (see Supplemental Information Table 1). Startle probes and US could occur pseudo-randomly from 5–25 seconds after entering a room with the limitation that there had to be 5 second between each event. Noise probes in the hallway occurred 5–10 second after entry.

### Valence and Arousal Ratings

Valence and arousal ratings were obtained using self-assessment manikin scales^50^ after freely navigating the rooms with a game controller but before context conditioning, and again after context conditioning. The arousal scale ranges 1 = extremely negative to 10 = extremely positive. The valence scale ranges 1 = extremely calm to 10 = extremely excited.

### Retrospective shock estimation and contingency awareness

Participants were asked to estimate the number of shocks they had received in the blue and yellow room and to estimate the percentage of times that they received a shock when they were in the blue and yellow room [see ref. 46].

### Post-experimental episodic memory test

An exploratory test of episodic memory consisted of 18 multiple-choice questions for each room (36 questions total). Questions probed memory on whether particular items were in a room, and the number, colour, and position of certain items. For the yellow room for example, we asked: “*What color was the rug below the circular table to the right as you entered the room*? *a*) *black*, *b*) *red*, *c*) *green*, *d*) *blue”*.

### iVR experience questionnaire

Participants indicated how they felt during the context conditioning task: “I felt no discomfort”, “I was a tiny bit uncomfortable, but not too bad”, “I was slightly uncomfortable”, “I was moderately uncomfortable and slightly nauseous”, “I was very uncomfortable and very nauseous”. They also indicated their experience using Virtual Reality technology and playing video games in general.

### Inventories and anxiety questionnaires

Participants completed the State-Trait Anxiety Inventory^51^, Intolerance of Uncertainty Scale^52^, Berkman-Syme Social Network Index^53^, and Childhood Trauma Questionnaire - short form^54^ at the conclusion of the study.

### Procedures

Upon arrival to the study (see Fig. 1 for study timeline), participants were given a brief overview of the study that included information about the Oculus Rift headset and a general description of the conditioning task. Following informed consent, participants were explained how to use the video game controller and asked to freely navigate an ‘infinite space’. They were then fitted with the Oculus Rift goggles and allowed to explore the rooms and hallway for 2 minutes to encourage pre-exposure to the contexts prior to context conditioning. Following free navigation the Oculus Rift goggles were removed and participants rated the rooms on dimensions of valence and arousal.

Shock electrodes were then attached and intensity was individually calibrated, followed by placement of EMG and SCR electrodes. Participants received headphones and were instructed that they would hear noise probes that would be loud but not uncomfortable and were presented with 9 startle probes to allow habituation of startle responses. Participants were then presented with 2D printed images of the rooms and the hallway and given the following instructions:

*“We will now start the task. You will not be able to move yourself but will move on a track. When you are in either the blue or yellow room, you may receive electrical shocks. Note that there is a relationship between the rooms and the shocks. When you are in the hallway you will not receive any shocks. In addition you will hear noise bursts throughout the task. To make sure you understood the instructions, can you tell me what the two rooms are? Also, in which rooms can you get shocks? It is important for the study that you try to stay as still as possible during the study and sit straight up in your chair. Try to pay attention to the relationship between the rooms and the shocks. The task will start in the hallway”.*

We choose to use a version of the widely used semi-instructed conditioning procedure to make sure that participants paid attention to the rooms and shocks to aid differential conditioning. Critically, we did not inform the participants about the exact contingency between the rooms and shocks, which they thus had to learn from reinforcement. Results from our startle and SCR measures (see below) confirm that participants learned the relationship between the contexts and shocks from reinforcement, *not* instruction.

After free navigation, shock calibration, electrode and headphone placement, startle habituation, and instructions, participants were again fitted with the Oculus Rift goggles and the task commenced . Note, participants were explicitly told that there was a relationship between the rooms and the shocks but not which room would be associated with shocks, which they had to learn through experience, because at pilot testing some participants verbalized an illusory correlation between an idiosyncratic behaviour they happened to be doing at the time of the shock (e.g., an object they happened to be looking at). This raised a potential confound in the interpretation of context-conditioning per se. The iVR context conditioning task lasted for 15 minutes. After completion of the task, subjects completed the valence and arousal ratings, shock contingency estimate, episodic memory test, VR experience questionnaire, inventories and anxiety questionnaires, and compensated $20.

### Statistics

Statistical analyses were performed in SPSS (IBM SPSS Statistics Inc.). Dependent measures were submitted to repeated measure ANOVAs and statistics were Greenhouse-Geisser or Huyn-Feldt corrected for non-sphericity when appropriate. Valence and Arousal ratings were subjected to a time (before, after) × context (CTX+, CTX−) 2 × 2 repeated measures ANOVA. Startle and SCR responses were subjected to a phase (early, late) x context (CTX+, CTX−, Hallway) 2 × 3 repeated measures ANOVAs. Significant findings from ANOVAs were followed up by paired- and independent samples t-tests. Means ± s.e.m are provided where relevant unless otherwise indicated.

## Results

We predicted that context conditioning in iVR would result in the acquisition of subjective and physiological threat responses and explicit threat memory.

### iVR tolerance

Participants on average tolerated the immersive Virtual Reality paradigm well. No participants dropped-out of the study or complained of motion sickness during the task. At the conclusion of the task, three participants (out of 22) indicated: “I felt no discomfort”; six indicated: “I was a tiny bit uncomfortable, but not too bad”; five indicated: “I was slightly uncomfortable”; four indicated: “I was moderately uncomfortable and slightly nauseous”; and four participants indicated: “I was very uncomfortable and slightly nauseous.” No subjects reported: “I was very uncomfortable and very nauseous,” which was the most extreme option.

### Subjective valence and arousal ratings

**Figure 2:**
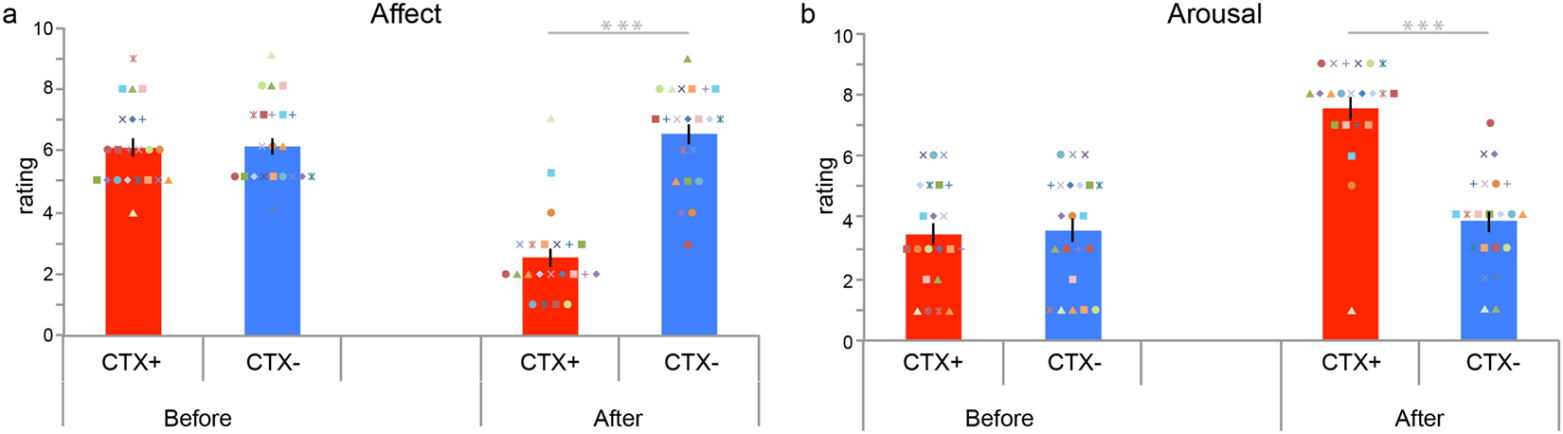
Context conditioning in iVR results in acquisition of subjective threat. Bar plots reflecting mean affect and arousal ratings before and after context conditioning for the threat (CTX+, red) and safe context (CTX−, blue). Context conditioning resulted in more negative affect ratings (**a**) and higher arousal ratings (**b**) for the threat context but did not affect ratings for the safe context. Error bars = s.e.m. Coloured geometrical shapes represent individual data-points. ***p < 0.001.

Context conditioning resulted in the acquisition of conditioned subjective threat memory. Before and after the context conditioning task participants rated the CTX+ and CTX− room on valence (negative-positive) and arousal (calm-excited) on a 9-point Likert scale (Fig. 2). Valence ratings changed from before to after context conditioning (time × context: F_(1,21)_ = 50.540, p < 0.001, ***η*** ^***2***^_***p***_ = 0.706. See Supplementary Information for full results of ANOVAs, t-tests, and descriptive statistics). Following context conditioning participants rated the context in which they had received electrical stimulation (CTX+) as more negative than the context in which they had never received electrical stimulation (CTX−) (t_(21)_ = −9.495, p < 0.001, Cohen’s d: 2.66). This effect was driven by participants rating the CTX+ as more negative following the context conditioning task then they did before the task (t_(21)_ = 8.549, p < 0.001, Cohen’s d: 2.583). Arousal ratings also changed from before to after context conditioning (time × context: F_(1,21)_ = 75.782, p < 0.001, ***η*** ^***2***^_***p***_ = 0.782). After conditioning participants rated the CTX+ context as more arousing than the CTX− context (t_(21)_ = 8.805, p < 0.001, Cohen’s d: 2.191) and this specifically stemmed from an increase in arousal ratings for the CTX+ context from before to after conditioning (t_(21)_ = −9.383, p < 0.001, Cohen’s d: 2.352). In sum, following conditioning participants reported the threat context to be more negative and more arousing than the control context.

### Eye-blink startle EMG responses

**Figure 3:**
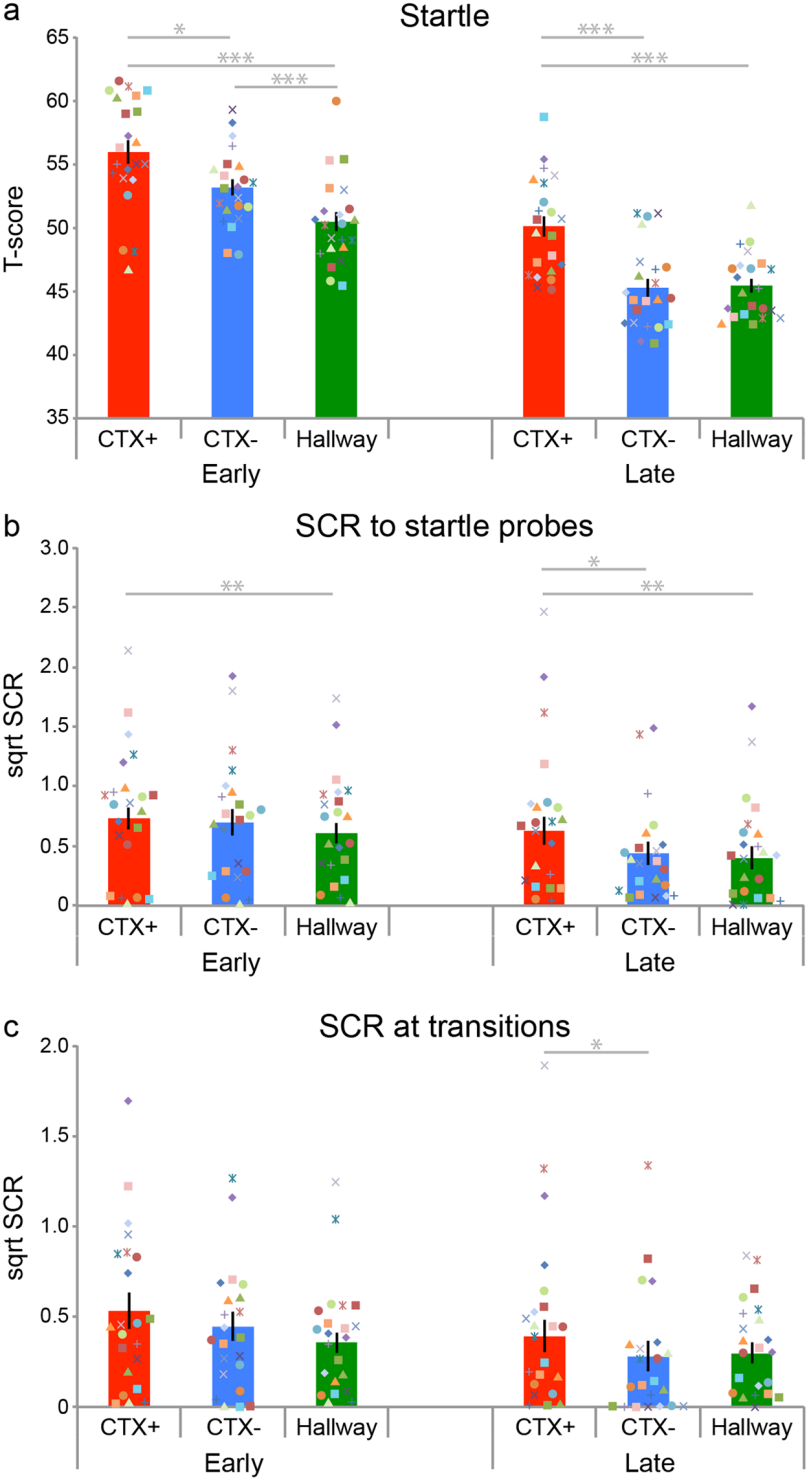
Context conditioning in iVR results in acquisition conditioned defensive responses. Bar plots reflecting mean startle and skin conductance responses in the first half (Early) and second half (Late) of the context-conditioning task for the threat (CTX+, red), safe context (CTX−, blue), and neutral context (Hallway, green). (**a**) Context conditioning resulted in greater electromyography responses (i.e. eye blink magnitude) to startle probes when participants traversed the threat context. (**b**) Context conditioning resulted in greater skin conductance responses (i.e. sweating) to startle probes when participants traversed the threat context. (**c**) Context conditioning resulted in greater skin conductance responses (i.e. sweating) when participants transitioned into the threat context. Error bars = s.e.m. Coloured geometrical shapes represent individual data-points. *p < 0.05, **p < 0.01, ***p < 0.001.

Context conditioning resulted in the acquisition of conditioned startle responses (phase × context: F_(2,42)_ = 2.469, p = 0.097, ***η*** ^***2***^_***p***_ = 0.105) (Fig. 3a). Critically, during the late phase of conditioning participants showed enhanced startle responses in the CTX+ context compared to the CTX− context (t_(21)_ = 4.078, p = 0.001, Cohen’s d: 1.374) and compared to the hallway (t_(21)_ = 4.775, p < 0.001, Cohen’s d: 1.444) but no difference in startle responses in the CTX− context compared to the hallway (t_(21)_ = −0.242, p = 0.811, Cohen’s d: 0.061). We found no evidence that EMG results were moderated by STAI-T, IUS, or childhood trauma scores, but we note that these scores were relatively low and homogeneous in this healthy undergraduate population. Context conditioning using iVR thus resulted in acquisition of conditioned eye-blink startle EMG responses to the threat context (for trial-by-trial analyses see Supplementary Information).

### Skin conductance responses

Although SCR is a common dependent measure of phasic responses in cue conditioning, it is less clear how to incorporate electrodermal measures of sympathetic arousal in context conditioning. One approach is to measure the tonic level of activity over the duration of the CTX+ as compared to the CTX−. However, startle probes occurred in each context and shocks were presented in CTX+ only, therefore complicating the analysis of tonic skin conductance levels. We therefore investigated SCR elicited by the startle probe and analysed SCRs upon the entrance of the CTX+, CTX−, and Hallway (Fig. 3b). Context conditioning resulted in the acquisition of conditioned SCRs to the startle probes (phase × context: F_(2,42)_ = 2.908, p = 0.066, ***η*** ^***2***^_***p***_ = 0.122). During the late phase of conditioning participants showed greater skin conductance responses to the startle probes while in CTX+ compared to CTX− (t_(21)_ = 2.706, p = 0.013, Cohen’s d: 0.522) and compared to the hallway (t_(21)_ = 3.361, p = 0.002, Cohen’s d: 0.415), but no difference in responses while in CTX− compared to the hallway (t_(21)_ = −0.455, p = 0.654, Cohen’s d: 0.072). We thus found a potentiation of startle-evoked SCRs by learned threat.

The potentiation of SCR (or eye-blink startle EMG, for that matter) under threat reflects a combination of anticipatory responses to threat (i.e. the context) with defensive responses to an intrinsically aversive stimulus (i.e. the startle probe). To obtain an estimate of anticipatory responses in isolation we assessed skin conductance responses as participants transitioned between the rooms and the hallway (Fig. 3b). With context conditioning participants acquired conditioned SCRs at transition points (phase × context: F_(2,42)_ = 0.720, p = 0.033, ***η*** ^***2***^_***p***_ = 0.033) so that during the late phase participants showed greater responses upon transitioning into CTX+ than CTX− (t_(21)_ = 2.426, p = 0.024, Cohen’s d: 0.453), and hallway at trend (t_(21)_ = 1.852, p = 0.078, Cohen’s d: 0.285), but no difference between transitioning into CTX− compared to the hallway (t_(21)_ = −1.172, p = 0.254, Cohen’s d: 0.201).

Context conditioning using iVR thus resulted in acquisition of potentiated startle and skin conductance responses while in the threat context and acquisition of anticipatory skin conductance responses when transitioning into the threat context (for trial-by-trial analyses see Supplementary Information). We found no evidence that STAI-T, IUS, or childhood trauma scores moderated SCR results.

### Retrospective shock estimation and contingency awareness

**Figure 4:**
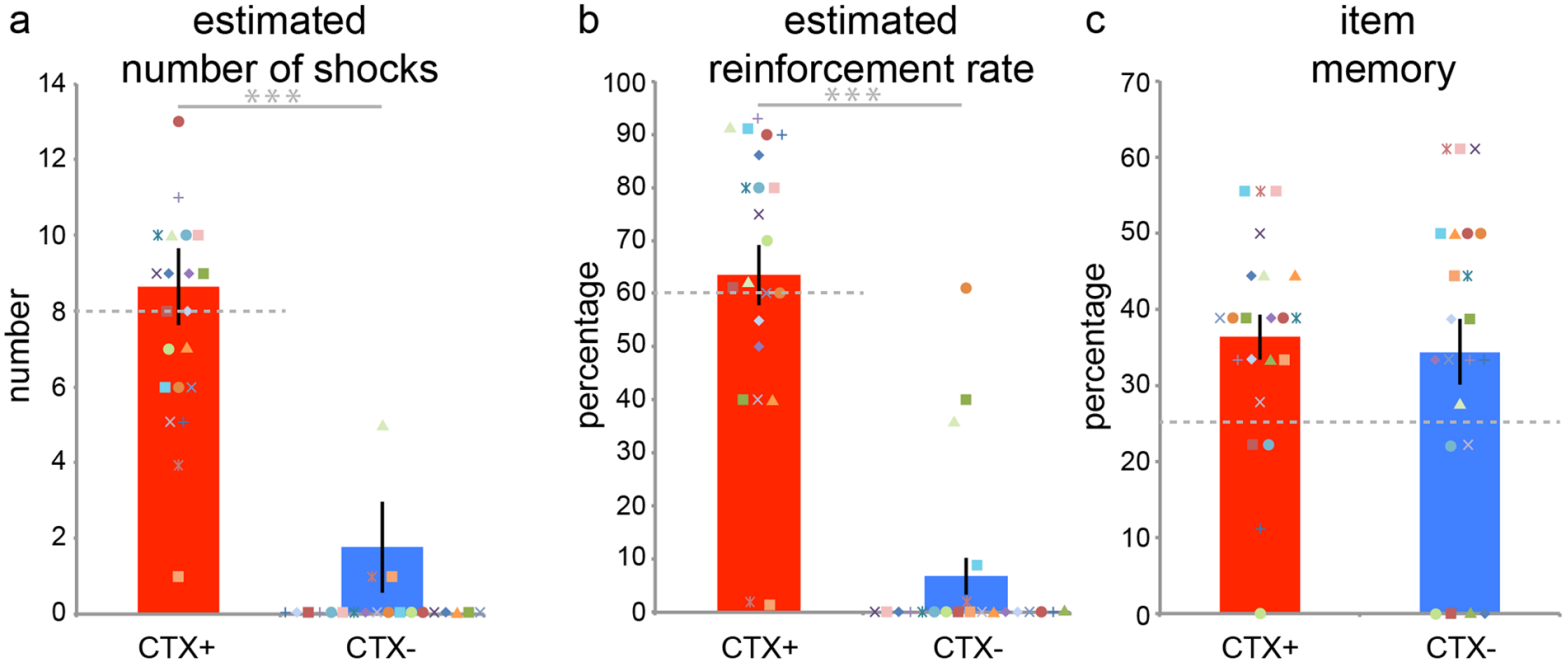
Context conditioning results in acquisition explicit threat memory. Bar plots reflecting mean estimated number of received shocks (**a**) and mean estimated reinforcement rate (**b**) for the threat (CTX+, red), safe context (CTX−, blue) tested at the end of the experiment. Dashed grey line indicates actual number of administered shocks (a: 8 shocks in CTX+ only) and actual reinforcement rate (**b**) 60% of CTX+ trials featured delivery of shock). Following iVR conditioning, participants accurately estimated having received more shocks in the CTX+ than CTX− and associated the CTX+ with a higher reinforcement rate then the CTX−. (**c**) Bar plots reflecting mean memory scores on four-alternative choice memory questionnaire testing memory for items that had been present in the CTX+ and CTX−. Dashed line indicates chance level (25%). Participants remembered items from both context above change there were no difference in item memory between contexts. Error bars = s.e.m. Coloured geometrical shapes represent individual data-points. ***p < 0.001.

Following context conditioning we asked participants to estimate the number of shocks they had received in CTX+ and CTX− and to estimate the percentage of times that being in each context resulted in shock, i.e. the reinforcement rate (Fig. 4a,b). Participants estimated having received more shocks in CTX+ than in CTX− (t(21) = 8.839, p < 0.001, Cohen’s d: 2.574) and their estimation was no different from the actual number of shocks received in CTX+ (one-sample t-test: t(21) = 0.632, p = 0.535, Cohen’s d: 0.135; actual number of shock = 8). Similarly, participants estimated a higher reinforcement rate for CTX+ than CTX− (t(21) = 8.063, p < 0.001, Cohen’s d: 2.574) that was no different from the actual reinforcement rate of 60% for CTX+ (t(21) = 0.616, p = 0.545, Cohen’s d: 1.131). Participants thus had explicit awareness of the association between the shocks and the threatening context, suggesting that the overall novelty of the iVR experience did not interfere with explicit knowledge of the CS-US contingencies.

### Item memory

At the end of the experiment we tested episodic memory for particular items that had been present in each room. As arousal is broadly associated with enhancements of episodic memory^55^, we reasoned that subjects might show differences in memory for items they encountered in the CTX+ versus CTX−. Memory performance overall was above chance level (four-alternative choice questionnaire = 25%) for CTX+ (t(21) = 3.864, p = 0.001, Cohen’s d: 0.824) and CTX− (t(21) = 2.187, p = 0.040, Cohen’s d: 0.466). However, there was no difference in memory for objects that had been present in the CTX+ compared to the CTX− (t(21) = 0.598, p = 0.557, Cohen’s d: 0.112; CTX+: 36.363, 2.941, CTX−: 34.343, 4.273). Thus, we found no evidence that the context conditioning task had an effect on episodic item memory.

## Discussion

The goal of this study was to develop a reliable novel context conditioning paradigm using a commercially available iVR headset and a free cross-platform game engine. Our results show that our iVR context conditioning protocol was well tolerated and resulted in reliable acquisition of subjective threat (arousal and valence measures), threat-conditioned defensive responses (EMG startle responses, and skin conductance responses to startle probes and in anticipation to transitioning into the threatening context) as well as explicit threat memory (knowledge of where and approximately how many shocks were given). These results add to a small body of literature of context conditioning in VR (see Table 1), and show iVR to be an effective and accessible tool to investigate contextual processes in humans [see ref. 56].

Development of readily available iVR context conditioning protocols provide a critical step toward bridging a long-standing translational gap between rodent and human research on the role of contextual processes. In rodents, context conditioning protocols provides a wealth of insight into contextual processes in learned threat^7, 9–12^. The use of iVR now allows investigators to ‘place’ human participants in different environments while maintaining experimental control, akin to context conditioning research with rodents. An advantage of iVR context conditioning protocols is that these can easily be adjusted to address a variety of research questions; for example the role of context representations in extinction, generalization^57^, and renewal of threat responses^58^, the role of context as an occasion setter, or the contribution of context to avoidance^59^. At the same time, human protocols can extend rodent research by including measures of explicit memory and subjective emotions like the feeling of fear and anxiety for discussion [see ref. 60].

To understand the neural mechanisms underlying contextual threat learning in humans, future studies can augment iVR context conditioning protocols for combination with electroencephalography, near infrared spectroscopy, or transcranial magnetic stimulation. However, the ability of the former methods to study the deep brain structures critical to contextual threat processes - like the hippocampal complex - is limited. Functional magnetic resonance imaging (fMRI) can be used to study such deep brain structures, but commercially available iVR head-mounted displays are currently not MR compatible and MR compatible VR displays are costly. Furthermore, head-movement in fMRI induces motion artefacts and therefore one of the defining immersive functionalities of iVR displays cannot be utilized during fMRI. One solution is to pre-expose participants to iVR contexts with motion prior to fMRI and study effects of contextual representations without motion during fMRI. Ongoing development of eye-tracking iVR head-mounted displays will also be useful to reduce the need for head motion to induce the sense of immersion and can also be utilized to study neural processes.

A second gap bridged by the development of iVR protocols is between laboratory studies and real-world experiences implicated in the development of emotional disorders. Future protocols should assess includes factors of emotional experiences that affect learning, such as threat intensity and egocentric distance to threat stimuli^39, 61, 62^. In the current study we report data from 22 healthy young participants (a sample size consistent with the extant human fear conditioning literature), and we therefore note the appropriate caution while discussing these results and hope to see attempts to replicate from other laboratories and in other populations. Importantly, as the iVR protocol was well tolerated it should be possible to assess contextual conditioning in a variety of populations including psychiatric patients. VR is increasingly being used as a treatment method for psychiatric disorders, such as posttraumatic stress disorder^63–65^. VR exposure therapy is an effective and validated form of treatment that is especially beneficial in situations where *in vivo* exposure is impractical, impossible, or unlikely to be tolerated^66, 67^. Yet, as most of the research on VR exposure is rightfully focused on clinical outcomes, it remains unclear how virtual experiences precisely engage the learning mechanisms underlying threat and safety learning. One recent investigation^68^ provides an example of incorporating recent advances in the neurobiology of learning and memory to a VR exposure situation. In this study participants with a fear of flying underwent VR exposure treatment 10 minutes after a phobia-reminder, a time window that has been shown in humans and animals to prevent the return of fear responses^69, 70^. Although the results were mixed, it provides an example of bringing knowledge from the domain of associative learning theory and the neurobiology of learning and memory to optimize VR treatment methods for anxiety- and stress-related disorders.

A limitation to the present study is that we did not allow volunteers to actively control their first-person movement in the virtual environment. We did so to limit the occurrence of motion sickness, to prevent problems novices had using a game controller, and to reduce distraction. However, this could have limited the full immersive experience. Unfortunately we did not include a subjective measure of immersion so we cannot assess the effect of our manipulations on the immersive experience of participants, a limitation future studies should address. Research in rodents suggest that self-movement can affect processes that underlie the formation of spatial representations [see e.g. ref. 71]. We did include a pre-exposure phase during which volunteers could control first-person movement to increase the immersive experience and to allow the initial formation of a representation of the spatial environment. In future studies, more extensive pre-exposure might circumvent navigation and distraction issues and could potentially allow self-control of movement throughout the paradigm. Furthermore, as commercial VR technology advances the risk of motion sickness will be reduced. In addition, with the emergence of devices that allow hand and limb movement and directed sound to be integrated into the iVR environment we expect that the immersive experience will increase and that movement can also be taken as a readout of human threat memory [see e.g. refs 59, 72 and 73].

As fully immersive VR technology advances and becomes more accessible, it will gain use as an important tool to the study of psychology and cognitive neuroscience. One area that stands to gain massively is the study of contextual processes - an area that has lagged behind laboratory animal research. We are convinced that the availability of a validated iVR context conditioning protocol that is made freely available to the scientific community is a critical step toward bridging a translational gap between rodent and human research on the role of contextual processes in threat learning as well as providing a more ecologically valid experimental approach to studying contextual contributions to psychopathology, which may ultimately lead to more optimal treatment strategies for anxiety- and stress-related disorders. As computer technology advances to replicate the sensation of realistic environments, there are increasing opportunities to investigate how context modulates human learning, memory, and emotions.

## Electronic supplementary material

Supplemental Information
