# Supplementary material for: Context conditioning in humans using commercially available immersive Virtual Reality
Source: Sci Rep. 2017 Aug 17;7:8640. doi: 10.1038/s41598-017-08184-7 (PMC5561126; doi:10.1038/s41598-017-08184-7)
Supplement: Supplementary file 1 — Supplemental Information [file 41598_2017_8184_MOESM1_ESM.pdf]

Context conditioning in humans using commercially available immersive Virtual Reality

- Supplementary Information -

Marijn CW Kroes[1,2,+,*], Joseph E Dunsmoor[1,+], Wayne Mackey[1], Mason McClay[3], and Elizabeth A Phelps[1,2,4*]

[1]New York University, Department of Psychology, New York, NY 10003, USA; [2]New York University, Center for Neural Science, New York, NY 10003, USA; [3]Centre College, Department of Psychology, Danville, KY 40422, USA; [4]Nathan Kline Institute, Orangeburg, NY 10962, USA

[*]Corresponding Authors:

Marijn CW Kroes, Department of Psychology, 6 Washington Place Room 890, New York University, New York, NY 10003 USA. Marijn.kroes@nyu.edu.

Elizabeth A. Phelps, Department of Psychology, 6 Washington Place Room 890, New York University, New York, NY 10003 USA. liz.phelps@nyu.edu.

[+]These authors contributed equally to this work

Tables

Table 1: Pseudo-randomization startle probes and USs across paths.

Context	# Of startle probes	# Of USs	# Of traversals
CTX+	2	0	4
CTX+	2	1	3
CTX+	2	1	1
CTX+	1	2	2
CTX-	2	0	8
CTX-	1	0	2
Hallway	1	0	20

The presentation of the noise probes and USs were pseudo-randomized across paths.

Table 2: ANOVA results

Measure	Time*	Context	Time x context
Valance	$F_{(1,21)}$=39.659, p<0.001, eta=0.654	$F_{(1,21)}$=61.494, p<0.001, eta=0.745	$F_{(1,21)}$=50.540, p<0.001, eta=0.706
Arousal	$F_{(1,21)}$=32.451, p<0.001, eta=0.607	$F_{(1,21)}$=53.058, p<0.001, eta=0.716	$F_{(1,21)}$=75.782, p<0.001, eta=0.782
	**Phase****	**Context**	**Phase x Context**
Startle	$F_{(1,21)}$=70.649, p<0.001, eta=0.771	$F_{(2,42)}$=18.621, p<0.001, eta=0.470	$F_{(2,42)}$=2.469, p=0.097, eta=0.105
SCR to probe	$F_{(1,21)}$=9.323, p=0.006, eta=0.307	$F_{(1.524,31.996)}$=7.566, p=0.004, eta=0.265	$F_{(2,42)}$=2.908, p=0.066, eta=0.122
SCR to room entry	$F_{(1,21)}$=3.912, p=0.028, eta=0.157	$F_{(1,21)}$=2.032, p=0.169, eta=0.088	$F_{(2,42)}$=0.720, p=0.033, eta=0.033
	**Trial*****	**Context**	**Phase x Context**
Startle trial x trial	$F_{(12.525,250.501)}$=11.381, p<0.001, eta=0.363	$F_{(2,40)}$=19.840, p<0.001, eta=0.498	$F_{(34,680)}$=1.633, p=0.014, eta=0.075
SCR trial x trial	$F_{(7.851,157.013)}$=2.399, p=0.019, eta=0.107	$F_{(1.581,31.630)}$=7.881, p=0.003, eta=0.283	$F_{(34,680)}$=1.422, p=0.059, eta=0.066
SCR to room entry trial x trial	$F_{(6.088,115.672)}$=3.304, p=0.005, eta=0.148	$F_{(2,38)}$=4.693, p=0.015, eta=0.198	$F_{(5.886,111.830)}$=0.647, p=0.019, eta=0.033

** Time = before or after the context conditioning task*

*** Phase = early (1st half of the trials), late (2nd half of the trials)*

**** Trial = trial 1-18 for CTX+ and CTX-, trial 2-19 for Hallway.*

SCR = skin conductance responses

Eta = partial eta squared (η^2_p)

Table 3: T-tests

Measure					
	Before	After	Before vs. After		
	CTX+ vs CTX-	CTX+ vs CTX-	CTX+	CTX-	
Valance	$t_{(21)}$=-0.137, p=0.892	$t_{(21)}$=-9.495, p<0.001	$t_{(21)}$=8.549, p<0.001	$t_{(21)}$=-1.252, p=0.224	

Arousal	$t_{(21)}$=-0.680, p=0.504			$t_{(21)}$=8.805, p<0.001			$t_{(21)}$=-9.383, p<0.001	$t_{(21)}$=-0.610, p<0.548	
	Early			Late			Early vs. Late		
	CTX+ vs CTX-	CTX+ vs Hallway	CS- vs Hallway	CTX+ vs CTX-	CTX+ vs. Hallway	CS- vs Hallway	CTX+	CTX-	Hallway
Startle	$t_{(21)}$=2.277 p=0.033	$t_{(21)}$=4.260 p<0.001	$t_{(21)}$=4.260 p<0.001	$t_{(21)}$=4.078 p=0.001	$t_{(21)}$=4.775 p<0.001	$t_{(21)}$=-0.242 p=0.811	$t_{(21)}$=4.643 p<0.001	$t_{(21)}$=8.238 p<0.001	$t_{(21)}$=5.154 p<0.001
SCR to probe	$t_{(21)}$=1.182, p=0.251	$t_{(21)}$=3.037, p=0.006	$t_{(21)}$=1.218, p=0.237	$t_{(21)}$=2.706, p=0.013	$t_{(21)}$=3.361, p=0.002	$t_{(21)}$=-0.455, p=0.654	$t_{(21)}$=0.987, p=0.335	$_{(21)}$=3.203, p=0.004	$t_{(21)}$=3.009, p=0.007
SCR to room entry	$t_{(21)}$=1.612, p=0.122	$t_{(21)}$=1.580, p=0.129	$t_{(21)}$=0.494, p=0.627	$t_{(21)}$=2.426, p=0.024	$t_{(21)}$=1.852, p=0.078	$t_{(21)}$=-1.172, p=0.254	$t_{(21)}$=0.715, p=0.483	$t_{(21)}$=1.696, p=0.105	$t_{(21)}$=1.089, p=0.288
				After					
				CTX+ vs CTX-					
Estimated # of shocks				t(21)=8.839, p<0.001					
Estimated % reinforcement				T(21)=8.063, p<0.001					
Episodic memory				T(21)=0.598, p=0.557					

Table 4: means and standard errors of the mean

Measure	Before		After	

	CTX+	CTX-		CTX+	CTX-	
Valance	6.091±0.278	6.136±0.289		2.546±0.300	6.136±0.289	
Arousal	3.455±0.359	3.591±0.382		3.591±0.382	3.864±0.331	
	**Early**			**Late**		
	CTX+	CTX-	Hallway	CTX+	CTX-	Hallway
Startle	55.948±0.93	53.157±0.636	53.157±0.636	50.122±0.810	45.267±0.694	45.447±0.545
SCR to probe	0.803±0.116	0.721±0.110	0.654±0.096	0.724±0.132	0.435, ±0.086	0.465± 0.093
SCR to room entry	0.536±0.094	0.424±0.73	0.389±0.066	0.463±0.101	0.268±0.073	0.331±0.054
				**After**		
				CTX+	CTX-	
Estimated # of shocks				8.636±1.008	1.773±1.204	
Estimated % reinforcement				63.500±5.682	6.818±3.507	
Episodic memory				36.363±2.941	34.343±4.273	

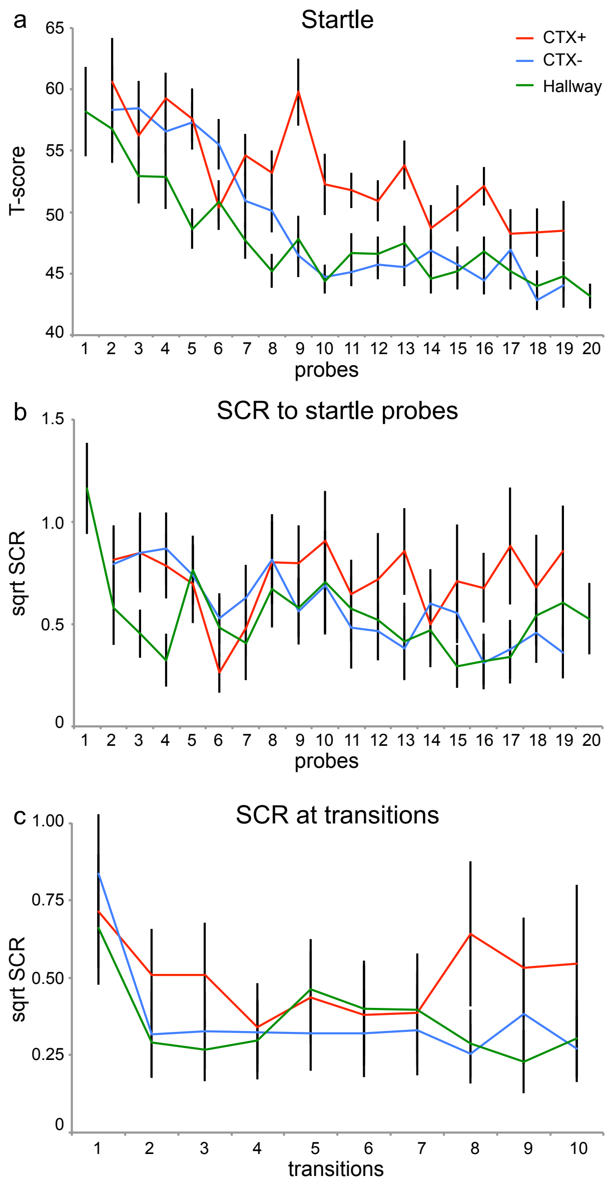

Figure 1: trial x trial startle and skin conductance responses. Line plots reflecting mean startle and skin conductance responses during the context-conditioning task for the threat (CTX+, red), safe context (CTX-, blue), and neutral context (Hallway, green). a) Context conditioning resulted in greater electromyography responses (i.e. eye blink magnitude) to startle probes when participants traversed the threat context. b) Context conditioning resulted in greater skin conductance responses (i.e. sweating) to startle probes when participants traversed the threat context. c) Context conditioning resulted in greater skin conductance responses (i.e. sweating) when participants transitioned into the threat context. Error bars = s.e.m.
